# Měnglà Virus VP40 Localizes to the Nucleus and Impedes the RIG-I Signaling Pathway

**DOI:** 10.3390/v17081082

**Published:** 2025-08-05

**Authors:** Joyce Sweeney Gibbons, Naveen Thakur, Emma Komers, Olivia A. Vogel, Poushali Chakraborty, JoAnn M. Tufariello, Christopher F. Basler

**Affiliations:** 1 Center for Microbial Pathogenesis, Institute for Biomedical Sciences, Georgia State University, Atlanta, GA 30303, USA; 2 Department of Microbiology, Icahn School of Medicine at Mount Sinai, New York, NY 10029, USA; naveen.thakur@mssm.edu (N.T.); emma.komers@mssm.edu (E.K.); olivia.vogel@mssm.edu (O.A.V.); joann.tufariello@mssm.edu (J.M.T.)

**Keywords:** Ebola virus, filovirus, importin, interferon, interferon regulatory factor 3 (IRF3), late-domain motif, Měnglà virus, Marburg virus, nuclear localization signal

## Abstract

Měnglà virus (MLAV) is a member of the genus *Dianlovirus* in the family *Filoviridae,* which also includes Ebola virus (EBOV) and Marburg virus (MARV). Whether MLAV poses a threat to human health is uncertain. However, the MLAV VP35 and VP40 proteins can impair IFNα/β gene expression and block IFNα/β-induced Jak-STAT signaling, respectively, suggesting the capacity to counteract human innate immune defenses. In this study, MLAV VP40 is demonstrated to impair the Sendai virus (SeV)-induced activation of the IFNβ promoter. Inhibition is independent of the MLAV VP40 PPPY late-domain motif that interacts with host proteins possessing WW-domains to promote viral budding. Similar IFNβ promoter inhibition was not detected for EBOV or MARV VP40. MLAV VP40 exhibited lesser capacity to inhibit TNFα activation of an NF-κB reporter gene. MLAV VP40 impaired IFNβ promoter activation by an over-expressed, constitutively active form of RIG-I and by the over-expressed IRF3 kinases TBK1 and IKKε. However, MLAV VP40 did not inhibit IFNβ promoter activation by constitutively active IRF3 5D. Consistent with these findings, MLAV VP40 inhibited SeV-induced IRF3 phosphorylation. Although IRF3 phosphorylation occurs in the cytoplasm, MLAV VP40 exhibits substantial nuclear localization, accumulating in foci in HeLa cell nuclei. In contrast, the VP40 of EBOV and MARV exhibited lower degrees of nuclear localization and did not accumulate in foci. MLAV VP40 interacts with importin alpha-1 (IMPα1), suggesting entry via the IMPα/IMPβ nuclear import pathway. Cumulatively, these data identify novel features that distinguish MLAV VP40 from its homologues in EBOV and MARV.

## 1. Introduction

Měnglà virus (MLAV) is a member of the genus *Dianlovirus,* in the family *Filoviridae* [1,2]. Other filovirus genera include *Cuevavirus,* which has a single member called Lloviu virus (LLOV), *Orthoebolavirus* and *Orthomarburgvirus* [3]. Members of the latter genera, including Ebola virus (EBOV) and Marburg virus (MARV), are zoonotic pathogens that cause outbreaks of severe disease in humans [4]. To date, MLAV has not been cultured, but its RNA was identified in China in a *Rousettus* bat’s liver [1]. Whether MLAV poses a threat to human health is unknown, but several MLAV-encoded proteins can function in human cells. For example, the glycoprotein can mediate viral entry, as assessed with a vesicular stomatitis virus-based pseudotype assay, and uses human NPC1 as a receptor [1]. The MLAV NP, VP35, VP30 and L proteins can replicate and transcribe a model viral genome; and the viral matrix protein VP40 can bud as virus-like particles in human cell lines [1,5]. These fundamental features of viral replication, entry, gene expression, genome replication and assembly suggest that MLAV will be able to infect humans, but the pathological consequences of infection remain unknown.

Filoviruses encode multiple mechanisms to counteract innate immune signaling pathways, and these inhibitory functions likely contribute to virulence [6]. Filoviral VP35 proteins suppress RIG-I-like receptor signaling and type I interferon (IFN) production, although the potency of suppression may vary between different viruses [7,8,9]. For EBOV VP35, this function has been demonstrated to be critical for virulence in mice, hamsters and non-human primates [10,11,12]. The VP24 proteins of the *Orthoebolavirus* and *Cuevavirus* genera, including EBOV, Bundibugyo virus (BDBV) and LLOV, block IFN-induced gene expression by preventing STAT1 nuclear translocation via interaction with select importin alpha (IMPα) nuclear transport proteins [13,14,15,16,17,18]. Inhibition of type I and type III IFN production has also been attributed to the VP24 proteins of EBOV and several other filoviruses [19]. MARV VP24 does not block type I IFN production but does bind the E3 ubiquitin ligase specificity factor Keap1 to promote the stabilization of transcription factor Nrf2, resulting in the upregulation of cytoprotective antioxidant response genes and the modulation of NF-κB [20,21,22,23]. MLAV VP24 appears to neither inhibit IFN-induced Jak-STAT signaling nor interact with Keap1 [5]. However, a different study reported some inhibition of IFN-induced gene expression by MLAV VP24 [18]. MARV VP40 inhibits IFN-induced Jak-STAT signaling by blocking activation of tyrosine kinase Jak1 [24]. This is a property distinct from that of EBOV VP40. However, similar to MARV VP40, MLAV VP40 inhibits IFN signaling [5].

Studies on EBOV and MARV demonstrated that VP40 also serves as the filovirus matrix protein and directs the budding of virus particles from infected cells [25,26]. Reflecting this function, expression of VP40 alone leads to budding and release of virus-like particles [25,27,28,29]. VP40s can adopt multiple oligomeric conformations to carry out their essential functions [30,31,32,33]. Structurally, the EBOV and MARV VP40s each fold into a distinct N-terminal domain (NTD) and a C-terminal domain (CTD) that are connected by a flexible linker [30,33,34]. Preceding the NTD is a disordered region of approximately 40 amino acid residues in EBOV VP40 and 35 amino acid residues in MARV VP40 [30,33]. Within the disordered N-terminus of VP40s are PPXY late-domain motifs that promote the release of infectious virions and enhance viral growth [25,35]. Late-domain motifs modulate filovirus budding through interactions with host proteins possessing WW-domains. The WW-domain proteins that promote VP40 budding include the E3 ubiquitin ligases Nedd4, Itch and WWP1 [25,36,37]. Negative regulators of budding also interact with the late-domain motif and include the WW-domain proteins BAG3, WWOX, YAP and TAZ [38,39,40]. In addition to promoting VP40 budding, late-domain-motif interactions can affect VP40 localization; for example, WWOX promotes EBOV VP40 nuclear localization [41].

Here, we sought to further characterize the apparent capacity of MLAV VP40 to inhibit virus-induced activation of the IFNβ promoter. The data identify features that distinguish MLAV VP40 from both EBOV and MARV VP40s. First, MLAV VP40 impairs RIG-I signaling by blocking phosphorylation and the activation of transcription factor IRF3 in a late-domain-motif-independent manner. Second, MLAV VP40 exhibits more robust nuclear accumulation than EBOV or MARV VP40 and accumulates in intranuclear foci. MLAV nuclear import correlates with its capacity to bind one of seven IMPα nuclear transport adaptor proteins, IMPα1, suggesting possible usage of the IMPα/IMPβ nuclear import pathway. Cumulatively, these findings increase the understanding of MLAV VP40–host interactions that may impact its ability to replicate and cause disease in vivo.

## 2. Materials and Methods

### 2.1. Cells, Viruses, Plasmids and Antibodies

HEK293T (ATCC), HeLa (ATCC) and Huh7 cells (a generous gift from the Gordan laboratory at the University of California at San Francisco) were maintained in Dulbecco’s Modified Eagle Medium, supplemented with 10% fetal bovine serum and cultured at 37 °C and 5% CO_2_. Sendai Virus Cantell (SeV) was grown in 10-day-old embryonating chicken eggs for forty-eight hours at 37 °C.

FLAG-tagged EBOV VP35, EBOV VP40, MARV VP40, MLAV VP40, RIG-I 2CARD, TBK1, IKKε, IRF3 and IRF3 5D expression plasmids and the IFNβ promoter–firefly luciferase reporter plasmid have been previously described [5,42,43,44,45]. The MLAV late-domain-motif (LD) mutant plasmid, which encodes a full-length protein with the late-domain-motif amino acid residues 16-PPPY-19 mutated to 16-AAAA-19, was generated for this study. The *Renilla* luciferase plasmid pRL-TK was purchased from Promega, Madison, WI, USA.

For Western blots, the following antibodies were used: Anti-FLAG (Sigma-Aldrich), anti-β-tubulin (Sigma Aldrich, Saint Louis, MO, USA), anti-glyceraldehyde-3-phosphate dehydrogenase (GAPDH), anti-total and Ser396 phosphorylated IRF3 (Cell Signaling Technology, Danvers, MA, USA) and HDAC2 (Cell Signaling Technology).

### 2.2. IFNβ and NF-κB Promoter Reporter Gene Assays

HEK293T cells (7 × 10^4^ per well of a 96-well plate) were co-transfected in triplicate, using TransIT^®^-LT1 (Mirus Bio, Madison, WI, USA), with 30 ng of an IFNβ promoter–firefly luciferase reporter or an NF-κB promoter–firefly luciferase plasmid, 30 ng of a plasmid constitutively expressing *Renilla* luciferase (pRL-TK, Promega), as well as 50, 16.7, 5.6 and 1.9 ng of VP35 and VP40 viral protein expression plasmids. Twenty-four hours post-transfection, cells were mock-treated, infected with SeV, or treated with TNFα (BD Pharmigen, 10 ng/mL). Twenty hours post-infection or treatment, cells were lysed and luciferase activity was measured using a Dual-Luciferase^®^ Reporter Assay System (Promega) per the manufacturer’s protocol. Firefly luciferase activity was normalized to *Renilla* luciferase activity. Normalized luciferase data was graphed as relative expression (fold change) versus the uninduced, empty vector control. Viral protein expression was confirmed by Western blot. Western blots were processed and quantified by using Bio-Rad Image Lab Software (version 6.1.0).

For assays that used the expression of RIG-I 2CARD, TBK1 or IKKε to activate the IFNβ promoter, 150 ng of the IFNβ-luciferase reporter, 150 ng of Renilla-luciferase plasmid and 50 ng of each inducer were used. Either 200 ng or 50 ng of each viral protein expression plasmid and up to 200 ng of empty vector were also transfected.

### 2.3. IRF3 Phosphorylation

HEK293T cells were transfected in 6-well plates using Lipofectamine 2000 Waltham, MA, USA). The amount of transfected IRF3 expression plasmid was 100 ng per well. Viral protein expression amounts were 80 ng and 2000 ng per well. Twenty-four hours post-transfection, the cells were mock-infected or SeV-infected. Subsequently, the cells were lysed in NP-40 buffer (50 mM Tris-HCl [pH 8.0], 280 mM NaCl, 0.5% NP-40) supplemented with cOmplete protease inhibitor cocktail (Roche, Basel, Switzerland) and PhosSTOP (Roche). Lysates were incubated for 10 min on ice and clarified for 10 min at 21,100× *g* at 4 °C. The phosphorylation status of the proteins was determined by Western blotting for total and Ser396-phosphorylated IRF3.

### 2.4. Immunofluorescence Assays

HeLa cells were seeded on coverslips at a density of 3 × 10^4^ cells per well of a 24-well plate for 24 h. The cells were transfected with 0.5 μg of FLAG-tagged plasmid DNA or empty vector control (pCAGGS) for 24 h. The cells were fixed with 4% paraformaldehyde in phosphate-buffered saline (PBS) for 10 min at room temperature. Fixed cells were washed twice with PBS-CM (phosphate-buffered saline, 1 mM CaCl_2_ and 1 mM MgCl_2_) and permeabilized for 10 min with 0.1% Triton X-100 in PBG (phosphate-buffered saline, 0.5% BSA and 0.15% glycine) at room temperature. After washing with PBS-CM, the cells were blocked with 4% goat serum in PBG for 1 h at room temperature. The cells were then incubated with anti-FLAG antibody conjugated to Alexa-Fluor 488 (Invitrogen, Waltham, MA, USA) in blocking buffer for 1 h at room temperature. Following incubation with the antibody, the cells were stained with Hoechst 33,342 trihydrochloride (Invitrogen) to stain the nuclei. The cells were washed three times with PBS-CM, and the coverslips were mounted with ProLong™ Glass Antifade Mountant (Invitrogen). To calculate the average nuclear fluorescence, a BioTek Cytation 10 Confocal Imaging Reader was used. A primary mask was set around the cell nucleus determined based on Hoechst nuclei staining. Average anti-FLAG Alexafluor488 fluorescence within the primary (nuclear) mask was recorded for each cell positive for anti-FLAG Alexafluor488 signal. To calculate the average cytoplasmic fluorescence, a secondary mask was created that began at the outline of the primary (nuclear) mask and extended outward until no anti-FLAG Alexafluor488 signal was detected above the set threshold for signal. Average anti-FLAG Alexafluor488 fluorescence within the boundaries of the secondary mask were recorded for each cell. These values were then used to calculate nuclear/cytoplasmic fluorescence (fn/c) per cell. Representative images of the same coverslips were acquired with the BioTek Cytation 10 Confocal Imaging Reader using the confocal microscope at 60x magnification.

### 2.5. Nuclear and Cytoplasmic Fractionation

HEK293T cells in 6-well plates were transfected with 500 ng of the indicated plasmids and amounts by using Lipofectamine 2000. At 24 h post-transfection, cytoplasmic and nuclear extracts were prepared according to the instructions of the NE-PER^®^ nuclear and cytoplasmic extraction kit (Thermo Fisher Scientific, Waltham, MA, USA).

### 2.6. Co-Immunoprecipitation Experiments

Transfection was performed in suspension using 1 × 10^6^ HEK293T cells per well of a 6-well plate. Lipofectamine2000 (Invitrogen) at a ratio of 1 µL Lipofectamine2000 to 1 µg DNA was used to transfect 2 µg FLAG-tagged MLAV VP40 and 2 µg HA-tagged IMPα per reaction. As a control for nonspecific binding, empty vector (pCAGGS) was used in place of HA-tagged IMPα. Twenty-four hours post-transfection, the cells were lysed in 1% NP-40 lysis buffer (50 mM Tris pH 7.5, 280 mM NaCl, 0.5% NP-40, 0.2 mM EDTA, 2 mM EGTA, 10% glycerol, protease inhibitor (cOmplete mini protease inhibitor tablets; Roche)). To precipitate HA-tagged IMPα, cell lysates were incubated with EZview anti-HA agarose affinity gel (Sigma Aldrich) for 1 h with rocking at 4 °C. Following incubation, beads were washed 4 times with 1% NP-40 buffer. Bound material was eluted from the beads with HA peptide (Sigma Aldrich) for 30 min with rocking at 4 °C. Whole-cell lysates and co-immunoprecipitation samples were run on a 4–12% Bolt polyacrylamide gel (Invitrogen) and transferred to a PVDF membrane (Sigma Aldrich). Following transfer, blots were incubated in blocking buffer (5% non-fat dry milk in 0.1%Tween20 (PBST)) for 1 h at room temperature. Blots were probed with the indicated antibodies, developed with Western Lightning plus ECL (Revvity, Waltham, MA USA) and imaged on the Bio-Rad ChemiDoc MP Imaging System.

## 3. Results

### 3.1. MLAV VP40 Inhibition of IFNβ Promoter Activation

**Figure 1:**
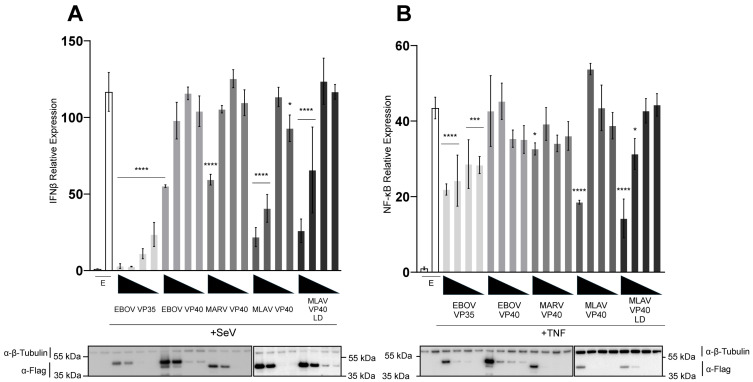
MLAV VP40 blocks Sendai virus-induced IFNβ promoter activation. HEK293T cells were co-transfected with an (**A**) IFNβ promoter–firefly luciferase reporter plasmid or (**B**) NF-κB promoter–firefly luciferase plasmid, a plasmid constitutively expressing *Renilla* luciferase reporter, and either empty vector (EV) or the specified FLAG-tagged viral proteins. Viral protein expression plasmids were transfected at 50, 16.7, 5.6 and 1.9 ng. Samples in (**A**) were mock-treated or infected with SeV, while samples in (**B**) were mock-treated or treated with TNFα to activate the promoter. Firefly luciferase activity was normalized to *Renilla* luciferase activity. Viral protein expression was confirmed by Western blot, as shown. Two gels were run and processed in parallel in order to include all samples. Transfections were performed in triplicate; error bars represent the SEM for the triplicate, and statistical significance was determined by performing a one-way ANOVA followed by Tukey’s multiple comparison test, compared to the SeV-infected or TNFα-treated empty vector control (*p* ≤ 0.05 = *, *p* < 0.001 = ***, *p* < 0.0001 = ****).

The capacity of FLAG-tagged MLAV VP40 to inhibit SeV infection-mediated activation of the IFNβ promoter was compared to FLAG-tagged EBOV and MARV VP40 by using a reporter gene assay in HEK293T cells. FLAG-EBOV VP35 was included as a known and potent inhibitor of this response (Figure 1A). SeV infection of empty vector-transfected cells resulted in a nearly 120-fold induction of the IFNβ promoter-directed luciferase expression as compared to uninfected, empty vector-transfected cells. This response was inhibited in a dose-dependent manner by each of the EBOV VP35 concentrations, as expected. Except for the highest concentration of MARV VP40, which very modestly decreased reporter gene expression, EBOV or MARV VP40 did not inhibit IFNβ promoter activation. MLAV VP40, in contrast, significantly inhibited reporter gene expression at the two highest concentrations tested (Figure 1A). Because MLAV VP40 has an apparent PPPY late-domain motif analogous to the MARV VP40 late-domain motif and because the EBOV and MARV VP40 late-domain motifs mediate interactions with host factors [35,37,38,39,40,41], a MLAV 16-PPPY-19 to 16-AAAA-19 late-domain (LD) mutant was tested and inhibited comparably to the wild type MLAV VP40. Cell lysates from triplicate samples were pooled and then assessed by Western blot, and relative levels were quantified (Supplementary Figure S1A).

To evaluate whether MLAV VP40 affects gene expression more broadly, reporter assays comparable to those described for IFNβ promoter inhibition were performed but with an NF-κB responsive promoter activated by TNFα. Under these conditions, VP35 expression only very modestly affected reporter gene expression, consistent with past observations that VP35 is not a potent inhibitor of NF-κB [46]. Neither EBOV nor MARV VP40 meaningfully inhibited reporter gene expression. MLAV VP40 and the MLAV VP40 LD mutant exhibited modest inhibition at the highest transfected amount (Figure 1B). Cell lysates from triplicate samples were pooled and then assessed by Western blot, and relative levels were quantified (Supplementary Figure S1B). Combined, these data demonstrate the capacity of MLAV VP40 to impair innate immune signaling in a late-domain-motif-independent manner. The stronger effects of MLAV VP40 on the IFNβ promoter assay suggest a potent disruption of RIG-I signaling pathway that is activated by SeV infection.

### 3.2. MLAV VP40 Prevents RLR Pathway Activation of IRF3

**Figure 2:**
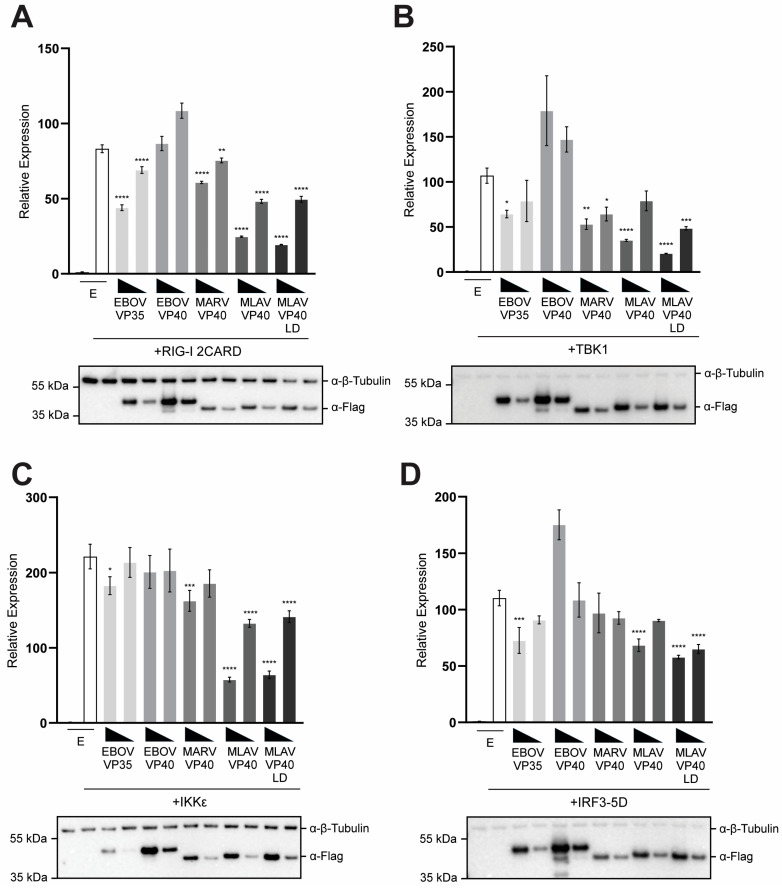
MLAV VP40 prevents RLR pathway activation of IRF3. HEK293T cells were transfected with an IFNβ promoter–firefly luciferase reporter plasmid, a constitutively expressing *Renilla* luciferase reporter plasmid, and either the empty vector (EV) or the specified FLAG-tagged viral proteins: EBOV VP40, MARV VP40, MLAV VP40 and MLAV VP40 LD. The expression plasmids for (**A**) RIG-I 2CARD, (**B**) TBK1, (**C**) IKKε or (**D**) IRF3 5D were also transfected. Firefly and *Renilla* luciferase activities were determined 18 h after transfection using a dual-luciferase assay. Induction was determined relative to the EV, uninduced samples. Transfections were performed in triplicate; error bars represent the SEM for the triplicate, and statistical significance was determined by performing a one-way ANOVA followed by Tukey’s multiple comparison test, compared to the empty vector, induced condition (*p* ≤ 0.05 = *, *p* < 0.01 = **, *p* < 0.001 = ***, *p* < 0.0001 = ****). For each transfection, Western blotting was performed with anti-FLAG and anti-β-tubulin antibodies, as indicated.

Upon sensing of activating RNA, RIG-I signals through the mitochondrial protein MAVS. This leads to the activation of the serine-threonine-kinases TBK1 or IKKε, which phosphorylate the transcription factor IRF3. Upon phosphorylation of IRF3 at specific serine residues, IRF3 migrates to the nucleus [47]. The combination of IRF3, NF-κB and AP-1 promotes activation of the IFNβ promoter [48]. The capacity of MLAV VP40 to inhibit IFNβ promoter activation by key components of this pathway was assessed by reporter gene assay. MLAV VP40 and MLAV VP40 LD inhibited IFNβ induction by over-expression of the CARD domains of RIG-I 2CARD, which acts as a constitutive activator of the IFNβ promoter [49]. Neither EBOV nor MARV VP40 inhibited induction in this setting (Figure 2A). Similar findings were made when the kinases TBK1 and IKKε, which are activated downstream of RIG-I and phosphorylate IRF3 and IRF7, were expressed. MLAV VP40 and MLAV VP40 LD inhibited IFNβ promoter activation by both TBK1 and IKKε (Figure 2B,C). Interestingly, while neither EBOV nor MARV VP40 inhibited the IFNβ promoter in the IKKε assay, MARV VP40 did exhibit some inhibitory activity in the TBK1 assay. When a constitutively active mutant of IRF3, IRF3 5D, which does not require phosphorylation by TBK1 or IKKε was tested, MLAV VP40 and MLAV VP40 LD exhibited only very modest inhibition, which was not substantially different from what was detected in EBOV VP40- or MARV VP40-expressing cells (Figure 2D). 

In each of these assays, EBOV VP35 exhibited only very modest inhibition, reflecting the fact that VP35 mainly inhibits the pathway upstream of TBK1 or IKKε kinase activation [45,50,51,52]. For all the assays in Figure 2, cell lysates from triplicate samples were pooled and then assessed by Western blot and relative levels were quantified (Supplementary Figure S2A–D). Combined, these findings suggest that MLAV VP40 likely blocks the TBK1 and IKKε phosphorylation of IRF3. However, when IRF3 phosphorylation is bypassed, inhibition is largely overcome.

### 3.3. MLAV VP40 Prevents Virus-Induced Phosphorylation of IRF3

**Figure 3:**
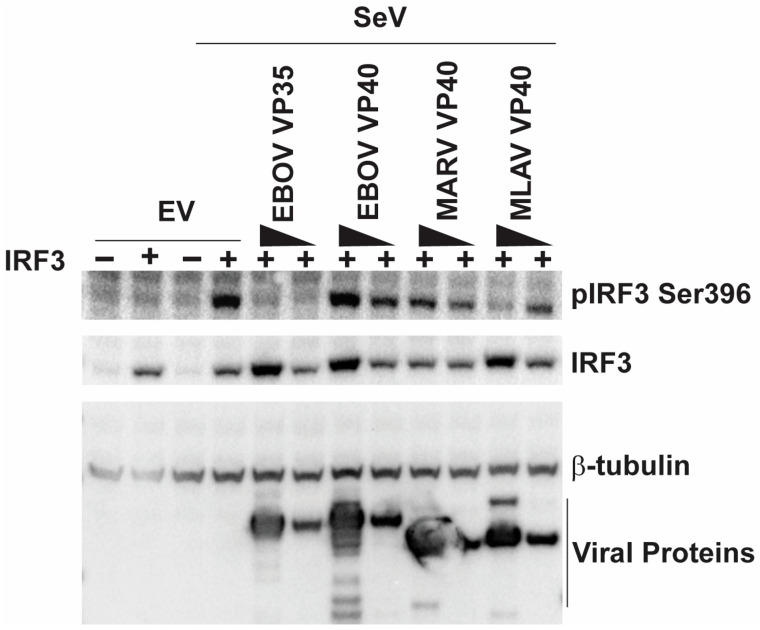
MLAV VP40 impairs SeV-induced IRF3 phosphorylation. Cells were transfected with empty vector (EV) or an IRF3 expression plasmid (indicated by +) and either of two concentrations of EBOV VP35, EBOV VP40, MARV VP40 or MLAV VP40 expression plasmids. The cells were then mock-infected or infected with SeV, as indicated. Four hours post-infection, whole-cell lysates were analyzed by Western blotting with anti-phosphoIRF3 (S396), anti-total IRF3, anti-FLAG and anti-β-tubulin antibodies. Relative levels of viral proteins, IRF3 and phospho-IRF3 were quantified (Supplementary Figure S3).

We next determined if MLAV VP40 can inhibit phosphorylation associated with IRF3 activation. HEK293T cells were either transfected with an empty vector, an IRF3 expression plasmid or with a combination of IRF3 and filovirus protein expression plasmids. One day post-transfection, cells were mock-infected or infected with SeV. The total levels of IRF3 and IRF3 phosphorylated at S396 were then assessed by Western blot and quantified (Figure 3 and Supplementary Figure S3A,B). In the absence of IRF3 over-expression, little evidence of phospho-IRF3 was detected. Upon infection with SeV, the phosphorylation of IRF3 was observed when IRF3 was over-expressed in the absence of filovirus proteins. As expected, EBOV VP35 strongly impaired IRF3 phosphorylation, whereas no significant inhibition was detected in EBOV VP40- or MARV VP40-expressing cells. MLAV VP40, however, did inhibit IRF3 phosphorylation in cells receiving the highest amount of VP40 expression plasmid. Because MARV VP40 and MLAV VP40 inhibit IFN-induced Jak-STAT signaling, a similar experiment was performed, except in the presence of the Jak kinase inhibitor ruxolitinib, in order to eliminate differences in IFN signaling as a variable between samples (Supplementary Figure S3C–E). The results mirrored those in Figure 3.

### 3.4. MLAV VP40 Localizes to the Nucleus

**Figure 4:**
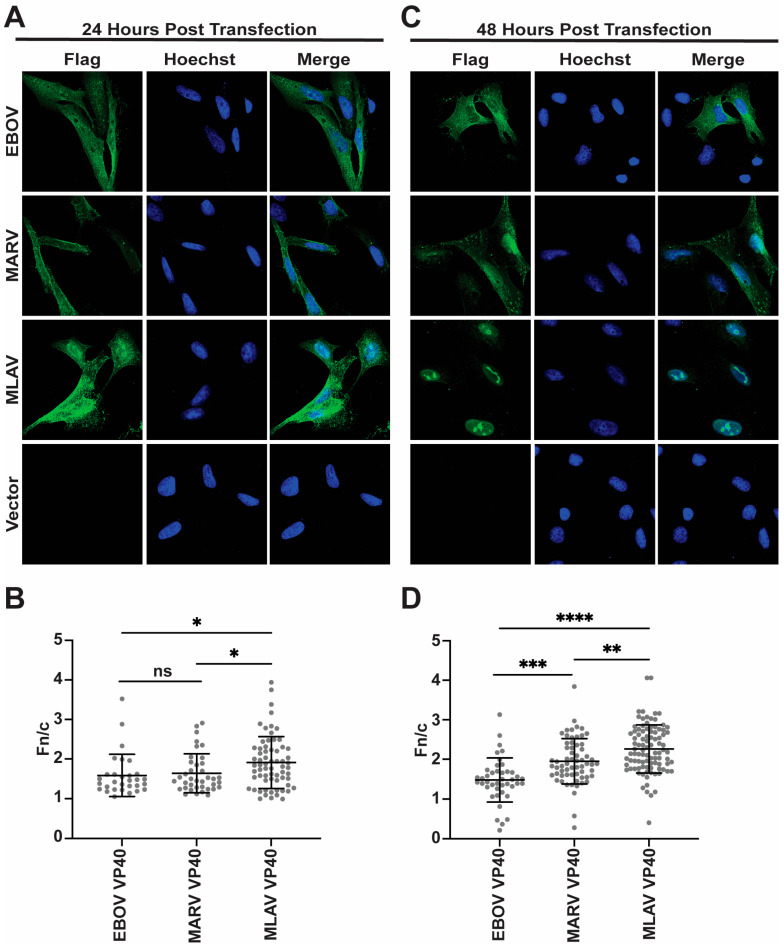
MLAV VP40 localizes to the nucleus. (**A**,**C**) Immunofluorescence confocal images to assess localization of FLAG-tagged EBOV, MARV and MLAV VP40 (green) at 24 h post-transfection (**A**) and 48 h post-transfection (**C**). (**A**,**C**) Empty vector (Vector) was included as a control. Hoechst (blue) staining indicates nuclei. (**B**,**D**) Average nuclear fluorescence over average cytoplasmic fluorescence for the indicated transfected FLAG-tagged viral proteins. (*p* ≤ 0.05 = *, *p* ≤ 0.01 = **, *p* ≤ 0.001 = ***, *p* ≤ 0.0001 = ****, *p* > 0.05 = ns).

**Figure 5:**
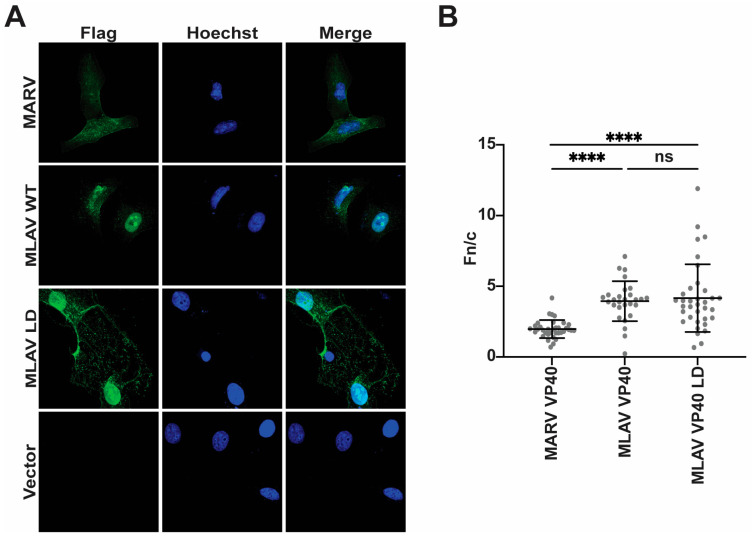
The mutation of the MLAV VP40 late-domain motif does not alter nuclear accumulation. (**A**) Immunofluorescence confocal images assessing localization of FLAG-tagged MARV, MLAV VP40 and MLAV VP40 LD mutant (green) at 48 h post-transfection. Empty vector (Vector) was included as a control. Hoechst (blue) staining indicates nuclei. (**B**) Average nuclear fluorescence over average cytoplasmic fluorescence (f/n) for the indicated transfected FLAG-tagged viral proteins. (*p* ≤ 0.0001 = ****, *p* > 0.05 = ns).

To further characterize MLAV VP40, its localization within HeLa cells was determined by using indirect immunofluorescence assays (IFA). For comparison, empty vector, FLAG-EBOV VP40 and FLAG-MARV VP40 were included. At 24 h post-transfection, nuclear localization was visible for each of the VP40s, but each retained a substantial cytoplasmic signal (Figure 4A). When the average nuclear versus cytoplasmic localization was quantified, MLAV exhibited the greatest level of nuclear accumulation (Figure 4B). By 48 h, EBOV and MARV VP40 exhibited somewhat increased nuclear localization, but cytoplasmic staining remained apparent. MLAV VP40 became more nuclear and accumulated in intranuclear foci with varying shapes (Figure 4C, Supplementary Figure S4). Cytoplasmic staining for MLAV VP40 became less apparent at this later time point. Quantification confirmed the increase in MLAV VP40 nuclear staining as compared to either EBOV or MARV VP40 (Figure 4D). To determine whether the late-domain motif affects MLAV VP40 nuclear localization, a similar experiment was performed that compared nuclear accumulation of MARV VP40, MLAV VP40 and MLAV VP40 LD at 48 h post-transfection. As before, MLAV VP40 was more nuclear than MARV VP40 (Figure 5A). MLAV VP40 and MLAV VP40 LD exhibited similar degrees of nuclear localization, and each displayed bright puncta in the nucleus (Figure 5B).

**Figure 6:**
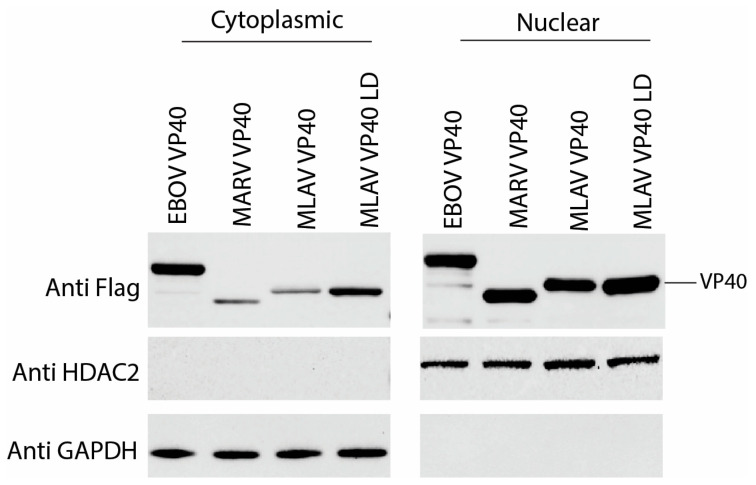
Nuclear and cytoplasmic VP40 levels detected by cell fractionation. Cells were transfected with expression plasmids for FLAG-tagged EBOV VP40, MARV VP40, MLAV VP40 and MLAV VP40 LD for 24 h. Viral proteins were detected with anti-FLAG antibody. HDAC2 expression served as a control for nuclear fractions and GAPDH expression served as a control for cytoplasmic fractions.

To further assess localization, FLAG-tagged MARV VP40, MLAV VP40 and MLAV VP40 LD were expressed in HEK293T cells. At 24 h post-transfection, the cells were separated into cytoplasmic and nuclear fractions, and these were analyzed by Western blot (Figure 6, Supplementary Figure S5A). GAPDH served as a cytoplasmic marker, and HDAC2 served as a nuclear marker. Each of the VP40 proteins was present in both the cytoplasmic and nuclear fractions. In this context, the percentage of total VP40 signal was most visibly greater in the nuclear fractions for MARV and MLAV VP40. Similar data were obtained when the cells were transfected for 48 h (Supplementary Figure S5B,C). These data confirm the capacity of the VP40s to traffic to the nucleus in a different cell type (Figure 6).

### 3.5. MLAV VP40 Interacts with Nuclear Import Adaptor IMPα1

**Figure 7:**
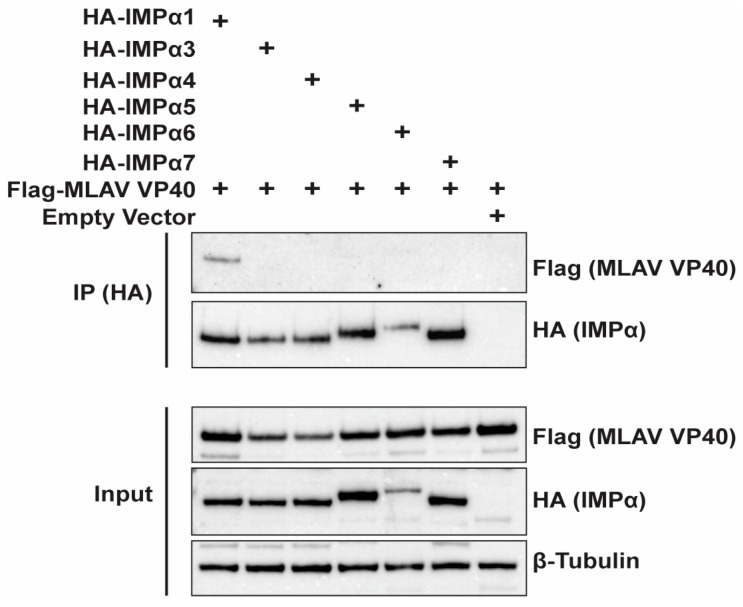
MLAV VP40 interacts with IMPα1. HEK293T cells were transfected with FLAG-tagged MLAV VP40 and the indicated HA-tagged IMPα constructs. As a control for nonspecific binding, empty vector was included. HA-tagged IMPα was immunoprecipitated with anti-HA beads and probed for interaction with FLAG-tagged MLAV VP40 via Western blot. β-tubulin expression served as a loading control for input samples.

To determine whether MLAV VP40 might enter the nucleus via the classical IMPα/IMPβ pathway, we co-transfected FLAG-MLAV VP40 with empty vector or HA-tagged members of the IMPα protein family. Anti-HA tag immunoprecipitations were then performed. MLAV VP40 detectably interacted only with IMPα1 (Figure 7). This suggests that MLAV VP40 may enter the nucleus, at least in part, through interaction with this nuclear transporter.

## 4. Discussion

Previous research has revealed both distinctions and commonalities in how filovirus proteins, specifically from MARV, EBOV and MLAV, modulate innate immune signaling. For instance, MLAV VP24 differs from its MARV counterpart, as it does not bind the host protein Keap1, thus failing to activate cellular antioxidant responses [5]. Similarly, unlike EBOV VP24, MLAV VP24 does not bind IMPα proteins, meaning that it does not block STAT1 nuclear import and, consequently, shows no detectable inhibition of IFNα/β responses [5]. At the same time, MLAV proteins share functional characteristics with those of other filoviruses. MLAV VP40 mirrors MARV VP40 in its ability to inhibit IFN-induced signaling, an activity linked to its impairment of the Jak1 function. Likewise, both MLAV and MARV VP35s share the capacity to inhibit the induction of the IFNβ promoter [5]. Prior studies also suggested that MLAV VP40 could inhibit IFNβ promoter activation in human- and bat-derived cell lines [5].

Here, we directly compared the potency of EBOV, MARV and MLAV VP40 proteins in inhibiting IFNβ promoter activation triggered by SeV infection, which primarily activates the RIG-I signaling pathway [53,54]. For reference, an alignment of the EBOV, MARV and MLAV VP40s is provided (Supplementary Figure S6). We included a MLAV VP40 late-domain-motif mutant in our analysis (Supplementary Figure S6). This is significant because the N-terminus of MLAV VP40 contains a PPPY motif, analogous to the MARV VP40 PPPY late-domain motif. In MARV and EBOV, PPXY late-domain motifs mediate interactions with host WW domain-containing proteins and are crucial for VP40 budding activity [28,35,38,39,40]. This motif has been implicated in specific host interactions, including with WW domain proteins such as YAP/TAZ, components of the Hippo signaling pathway known to regulate innate immune signaling, including IFNβ promoter activation [55]. Our data demonstrated that MLAV VP40 potently inhibited IFNβ promoter activation, and this function was independent of its late-domain-motif interactions. In contrast, under the experimental conditions, EBOV VP40 showed no inhibitory activity, and MARV VP40 exhibited only marginal inhibition, even at the highest concentration tested.

To determine whether the effects of MLAV VP40 are specific to the activation of the IFNβ promoter, we compared the effects of the VP40s on TNFα-mediated activation of an NF-κB-responsive promoter. In this context, we detected modest inhibition by MLAV VP40 and MLAV VP40 LD at the highest concentration tested. While the degree of inhibition was relatively minor, it nonetheless distinguished MLAV VP40 from EBOV and MARV VP40s, neither of which exhibited meaningful inhibition in this assay. Notably, EBOV VP35 also showed negligible inhibition of NF-κB activation. This result aligns with previous data indicating that VP35 does not directly inhibit NF-κB [46]. The more pronounced inhibition of the IFNβ promoter assay, compared to the NF-κB assay, supports our further investigation into the specific effects of MLAV VP40 on the RIG-I signaling pathway. However, given the demonstrable, albeit modest, inhibition observed in the NF-κB assay, whether MLAV VP40 may exert broader effects on innate immune signaling or global host gene expression warrants further investigation.

To gain further mechanistic insight into MLAV VP40′s IFNβ promoter inhibitory activities, we employed a standard approach involving the overexpression of key RIG-I signaling pathway components in the presence or absence of the putative inhibitor [56,57]. Upon overexpression, these transfected signaling proteins activate the pathway without an additional external stimulus. Utilizing this strategy, we demonstrated that MLAV VP40 effectively inhibited IFNβ promoter activation induced by the overexpression of constitutively active RIG-I, TBK1 or IKKε. The MLAV VP40LD mutant behaved almost identically, confirming that this inhibitory activity is independent of its late-domain motif. This pattern of inhibition strongly suggests that MLAV VP40 acts at or downstream of TBK1 and IKKε, potentially by preventing the phosphorylation of IRF3 by these kinases. Consistent with this hypothesis, we observed significantly less inhibition of IFNβ promoter activation when constitutively active IRF3 (IRF3 5D) was overexpressed. This indicates that MLAV VP40′s primary inhibitory activity occurs upstream of IRF3 nuclear translocation. The modest inhibition observed with EBOV VP35 in these assays is likely a reflection of its known mechanism, which involves preventing the initial activation of RIG-I itself [8,50,51,52]. Further supporting our model, MLAV VP40 demonstrated the capacity to impair IRF3 phosphorylation induced by SeV infection, although this inhibition was not as pronounced as that seen when EBOV VP35 was expressed. The overall picture emerging from these studies is that MLAV VP40 functions to prevent IRF3 phosphorylation, and that this is a key contributor to its inhibition of IFNβ production. While this appears to be a main mechanism, we cannot rule out that other aspects of the type I IFN induction pathways are also affected, particularly given the very modest but still detectable inhibition of IFNβ promoter activation when IRF3 5D was overexpressed.

While RIG-I-mediated sensing of viral RNA and the subsequent downstream signaling leading to IRF3 phosphorylation are well-established cytoplasmic events, the strong nuclear localization of MLAV VP40 when assessed by indirect immunofluorescence assay in HeLa cells was notable. This nuclear localization became particularly apparent at 48 h post-transfection of the MLAV expression plasmid. The nuclear MLAV VP40 concentrated within intranuclear structures of varying shapes that warrant further characterization. The MLAV VP40 LD mutant yielded similar results. Although we also detected nuclear EBOV and MARV VP40, the MLAV VP40 nuclear signal was notably more pronounced. In HEK293T cells, when localization was assessed by cell fractionation, all VP40s tested were again found to localize to the nucleus. Differences between HeLa and HEK293T cells could reflect the large nuclear-to-cytoplasmic volume ratio and the high levels of expression in the latter cells. Cumulatively, these data demonstrate that MLAV VP40 is nuclear and that in HeLa cells, it exhibits a distinct nuclear localization pattern, providing another distinction between MLAV VP40 and its EBOV and MARV counterparts, though its functional significance in the context of innate immune evasion or viral replication remains to be determined.

The nuclear trafficking of matrix (M) proteins is a common feature among non-segmented negative-sense RNA viruses. The vesicular stomatitis virus (VSV) M, for instance, localizes to the nucleus and nuclear pores, suppressing host transcription and nucleocytoplasmic transport [58,59,60,61]. The respiratory syncytial virus matrix protein also localizes to the nucleus and inhibits cellular transcription [62,63]. Similarly, the paramyxovirus M proteins enter the nucleus, with the nuclear trafficking of the Nipah virus M appearing essential for its budding [64,65]. The intranuclear Nipah and Hendra virus M localize to nucleoli and inhibit ribosomal RNA synthesis [64,66,67,68]. The integration of these nuclear M protein functions with their roles in viral assembly and release remains incompletely characterized.

The functional significance of filovirus VP40 nuclear localization is also poorly understood. Filoviral VP40 interacts with cellular membranes and WW-domain containing host factors to drive particle budding [28,69]. The Marburg virus (MARV) VP40 traffics from perinuclear regions to the plasma membrane via late endosomal membranes [70]. While primarily cytoplasmic and associated with nucleocapsids, intranuclear MARV VP40 has been noted [69]. The Ebola virus (EBOV) VP40 also localizes to the nucleus [71,72,73]. One study linked the nuclear EBOV VP40 to increased cell viability, altered cell cycling and elevated cyclin D1, further demonstrating its association with the cyclin D1 promoter via chromatin immunoprecipitation [74].

How MLAV VP40 traffics to the nucleus is also of interest. In the classical nuclear import pathway, a cargo protein’s nuclear localization signal (NLS) typically binds to one of seven IMPα isoforms [75]. This complex then associates with importin β1, which facilitates its transport through the nuclear pore [75]. Given this mechanism, we investigated MLAV VP40 interaction with all seven human IMPα isoforms using co-immunoprecipitation. This identified IMPα1 as an interactor, suggesting it mediates MLAV VP40 nuclear translocation. However, the exact nature of this interaction remains to be fully defined. Classical NLSs (cNLSs) that bind to IMPα proteins are classified as either monopartite, consisting of a single cluster of basic amino acids: K (K/R)X (K/R), or bipartite, featuring two clusters separated by 10–12 residues: R/X (X)_10–12_KRXK [76]. Analysis of the MLAV VP40 amino acid sequence did not reveal an identifiable classical nuclear localization signal using one NLS search program, cNLS mapper, with default settings [77]. However, the program PSORTII identified a sequence that conformed to what is termed pattern 7 [78]. This is a sequence that begins with P and is followed by a stretch of four residues, three of which are K or R. NLSs conforming to this definition have been demonstrated in the porcine reproductive and respiratory syndrome virus (PRRSV) nucleocapsid protein (N) (sequence PGKKNKK) and the Cullin E3 ligase VACM-1/CUL5 (sequence PKLKRQ) [79,80]. The predicted MLAV VP40 NLS is 72-PTEKGRK-78. Based on the MARV VP40 structure, this sequence is likely part of an exposed loop, which would make it readily accessible to nuclear transporters [33]. Although the precise arrangement of basic residues relative to the proline in MLAV VP40 differs from PRRSV N and VACM-1/CUL5, previous studies on PRRSV NLS revertants have shown that variations in the position and number of basic residues within this region can still enable nuclear import [79].

It is also possible that MLAV VP40 enters the nucleus by interacting with another NLS-bearing protein. For example, WW domain-containing oxidoreductase (WWOX) binds the EBOV VP40 PPXY motif [38]. Co-expression of WWOX increased EBOV VP40 nuclear localization [41], suggesting that WWOX might facilitate VP40 nuclear import via its own nuclear localization signal [41]. In our assays, the MLAV VP40 late-domain-motif mutant behaved the same as wildtype MLAV VP40, suggesting a WWOX independent mechanisms of nuclear import. The identification of the sequences that mediate MLAV VP40-IMPα1 interaction would enable the generation of loss-of-binding mutants to test the effects on MLAV VP40 localization and function.

Cumulatively, the studies described here identify novel functional features of MLAV VP40 that distinguish it from EBOV and MARV. How the properties described here will impact the outcome of infection will require the development of systems that enable the study of the viral replication cycle. A full-length viral genome sequence and functional minigenome systems have been described [81]. Therefore, the development of reverse genetics and viral lifecycle modeling systems analogous to those used for EBOV and MARV should be feasible. Such systems will also help address whether MLAV has pathogenic potential in humans.

## Data Availability

Data will be made available upon request following publication.

## Supplementary Materials

The following supporting information can be downloaded at: https://www.mdpi.com/article/10.3390/v17081082/s1, Figure S1: Relative expression levels of proteins from Figure 1 western blots.; Figure S2: Relative expression levels of proteins from Figure 2 western blots.; Figure S3: Relative expression levels of proteins from Figure 3.; Figure S4: Relative expression levels of proteins from Figure 3.; Figure S5: Cell fraction analysis.; Figure S6: Alignment of EBOV, MARV and MLAV VP40 amino acid sequences..

## Abbreviations

The following abbreviations are used in this manuscript:

BDBV	Bundibugyo virus
BSA	bovine serum albumin
CARD	caspase recruitment domain
CO_2_	carbon dioxide
CTD	carboxy terminal domain
EBOV	Ebola virus
GP	glycoprotein
IFA	immunofluorescence assay
IFN	interferon
IKKε	IκB kinase ε
IRF3	interferon regulatory factor 3
IRF7	interferon regulatory factor 7
Jak	Janus kinase
Keap1	Kelch-like ECH-associated protein 1
L	large protein
LD	late domain
LLOV	Lloviu virus
MARV	Marburg virus
MLAV	Měnglà virus
NP	nucleoprotein
Nrf2	nuclear factor erythroid 2-related factor 2
NTD	amino terminal domain
NPC1	Niemann-Pick disease type C1
PBS	phosphate buffered saline
RIG-I	retinoic acid-inducible gene I
SeV	Sendai virus
STAT	signal transducer and activator of transcription
VP24	viral protein of 24 kilodaltons
VP30	viral protein of 30 kilodaltons
VP35	viral protein of 35 kilodaltons
VP40	viral protein of 40 kilodaltons
WWOX	WW domain-containing oxidoreductase
