# Supplementary material for: Měnglà Virus VP40 Localizes to the Nucleus and Impedes the RIG-I Signaling Pathway
Source: Viruses. 2025 Aug 5;17(8):1082. doi: 10.3390/v17081082 (PMC12390687; doi:10.3390/v17081082)
Supplement: Supplementary file 1 [file viruses-17-01082-s001.zip › viruses-3774275-supplementary.pdf]

Figure 1A Left

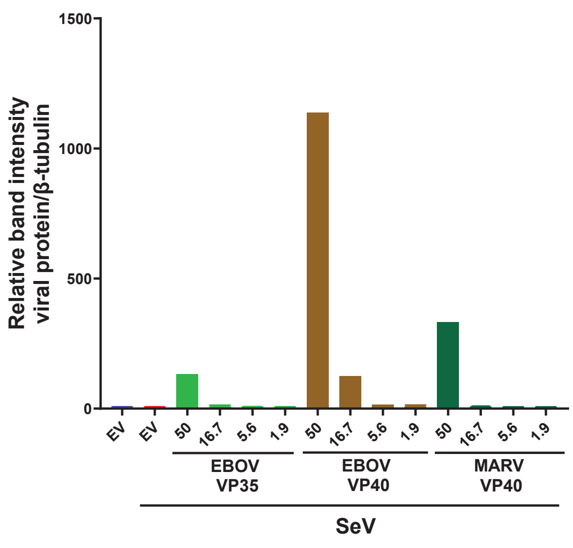

Figure 1A Right

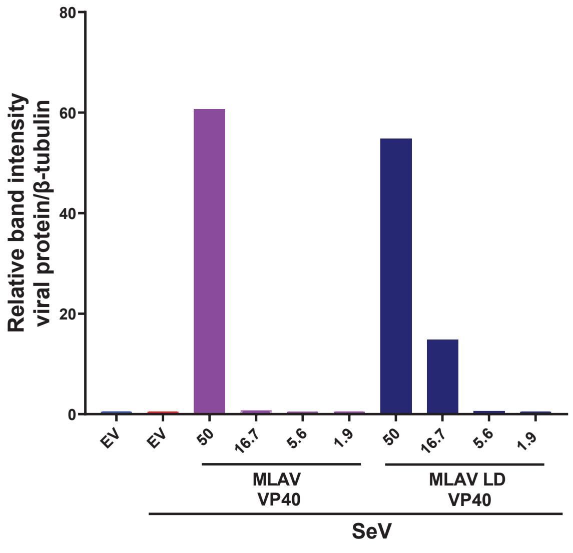

Figure 1B Left

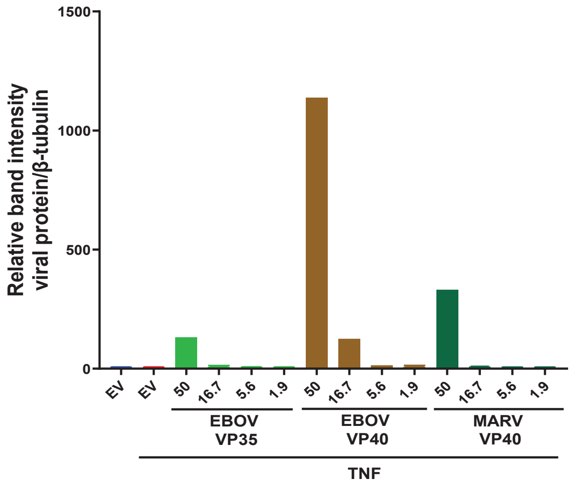

Figure 1B Right

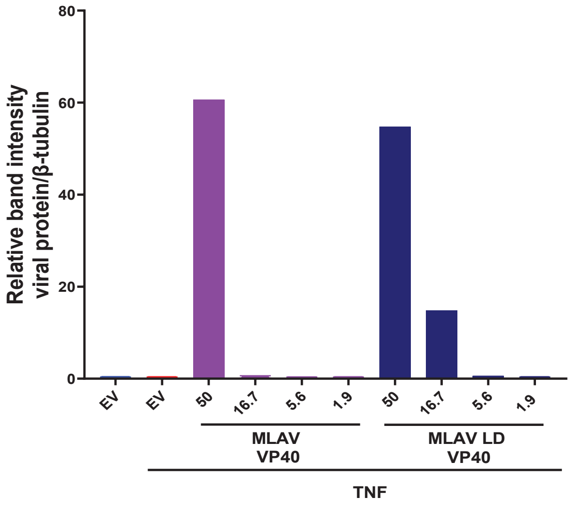

Supplemental Figure S1. Relative expression levels of proteins from Figure 1 western blots. The ratio of the chemiluminescence signal of each Flag-tagged protein to the intensity of the β-tubulin band from the same lane was determined. The resulting value was multiplied by 100 to express it as a percentage. Two graphs (Left and Right) are provided for each panel (A and B) because, due to the number of samples. two gels were run for each experiment.

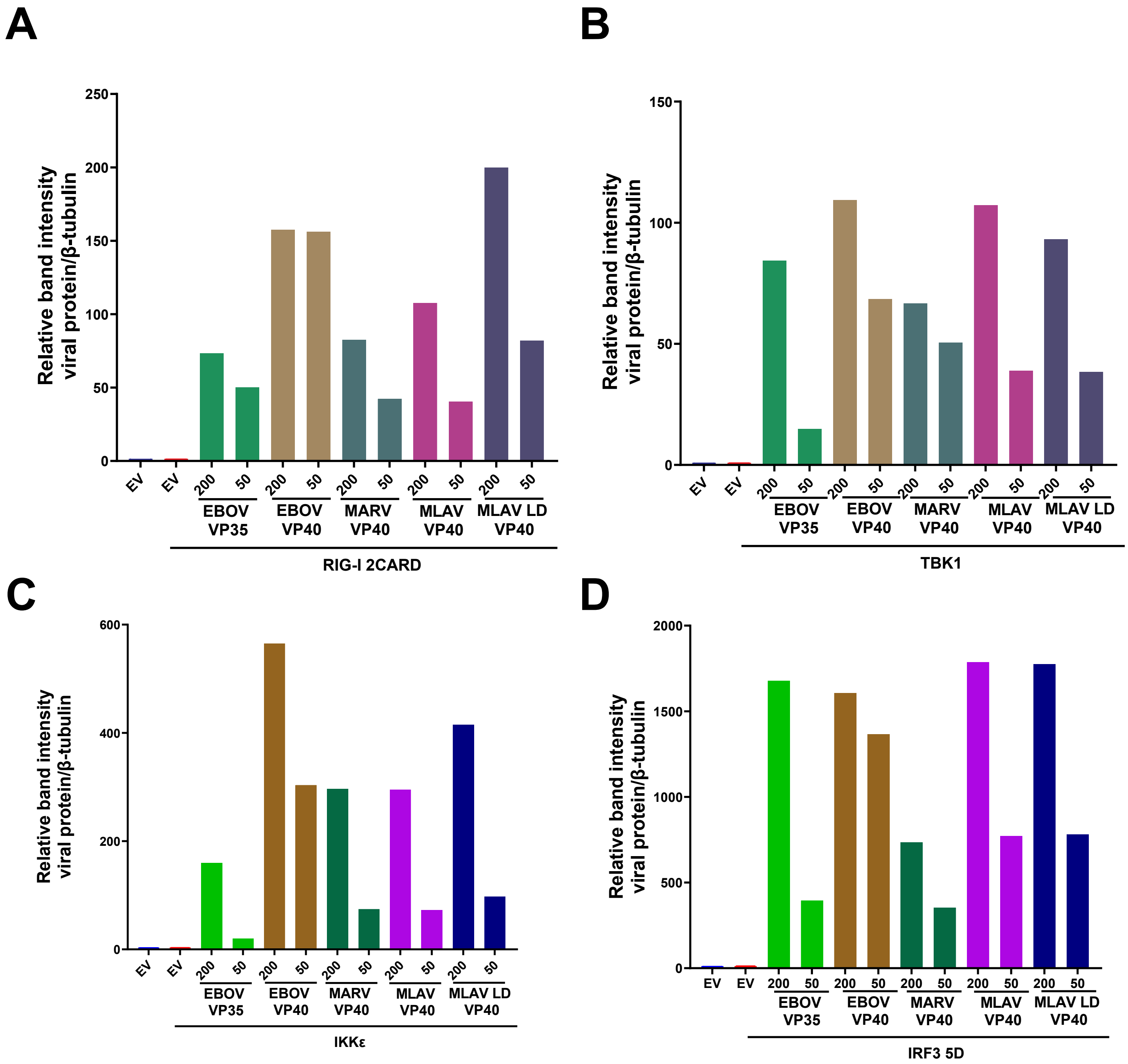

Supplemental Figure S2. Relative expression levels of proteins from Figure 2 western blots. The ratio of the chemiluminescence signal of each Flag-tagged protein to the intensity of the β-tubulin band from the same lane was determined. The resulting value was multiplied by 100 to express it as a percent-age.

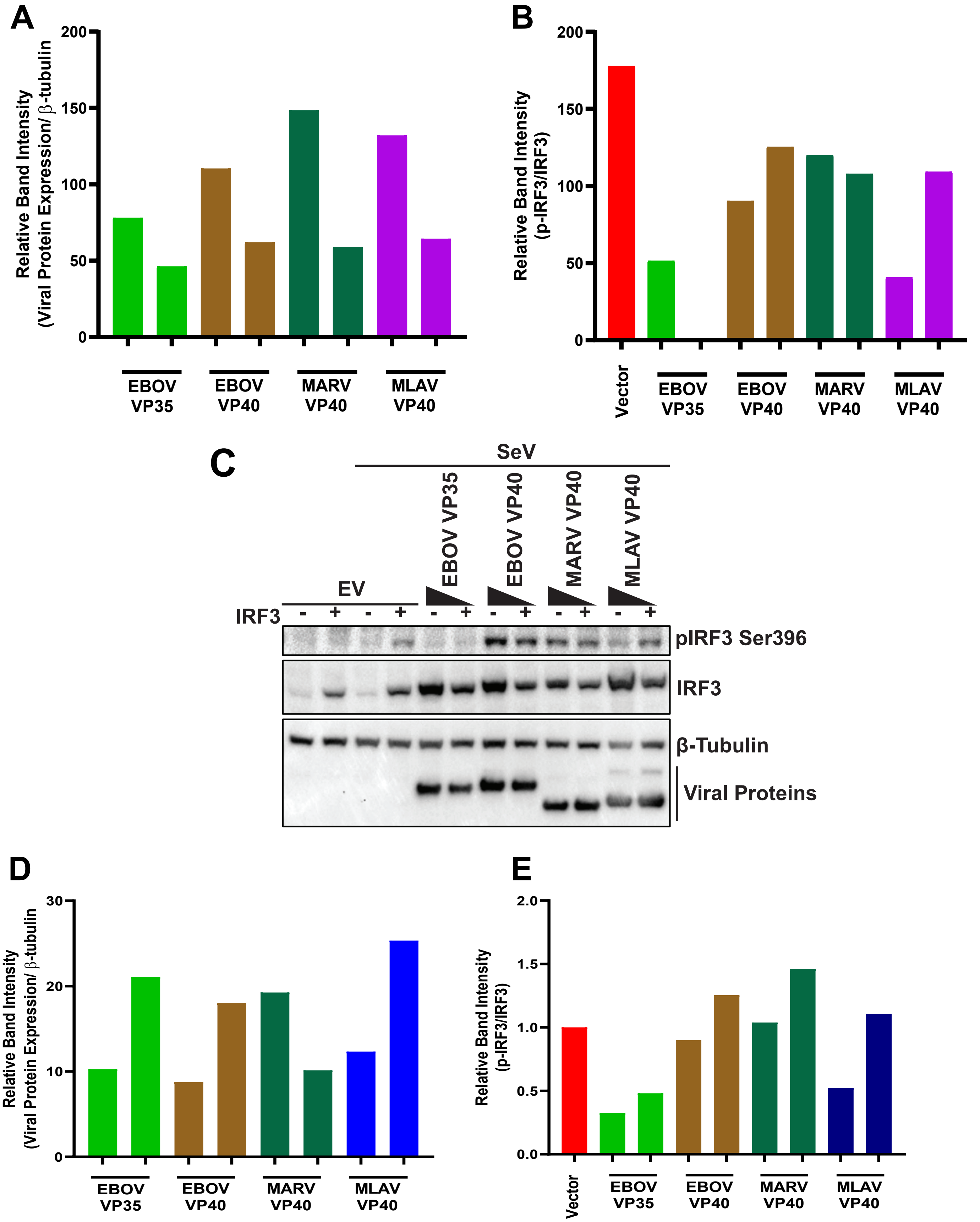

Supplemental Figure S3. Relative expression levels of proteins from Figure 3. A. The ratio of the chemiluminescence signal from Figure 3 of each Flag-tagged protein to the intensity of the β-tubulin band from the same lane was determined. Only lanes with transfected Flag-tagged viral proteins were included in this analysis. B. The ratio of the chemiluminescence signal of phospho-IRF3 to total IRF3 the same sample was determined. Only lanes that were transfected with IRF3 expression plasmid and that were SeV-infected were included in this analysis. The resulting value was multiplied by 100 to express it as a percentage. C. Western blots monitoring total and phospho-IRF3 levels and levels of Flag-tagged viral proteins. The methods were the same as for Figure 3 except that all samples were treated with Jak kinase inhibitor Ruxolitinib. D and E. Quantification of the samples from panel C, performed as described for panels A and B.

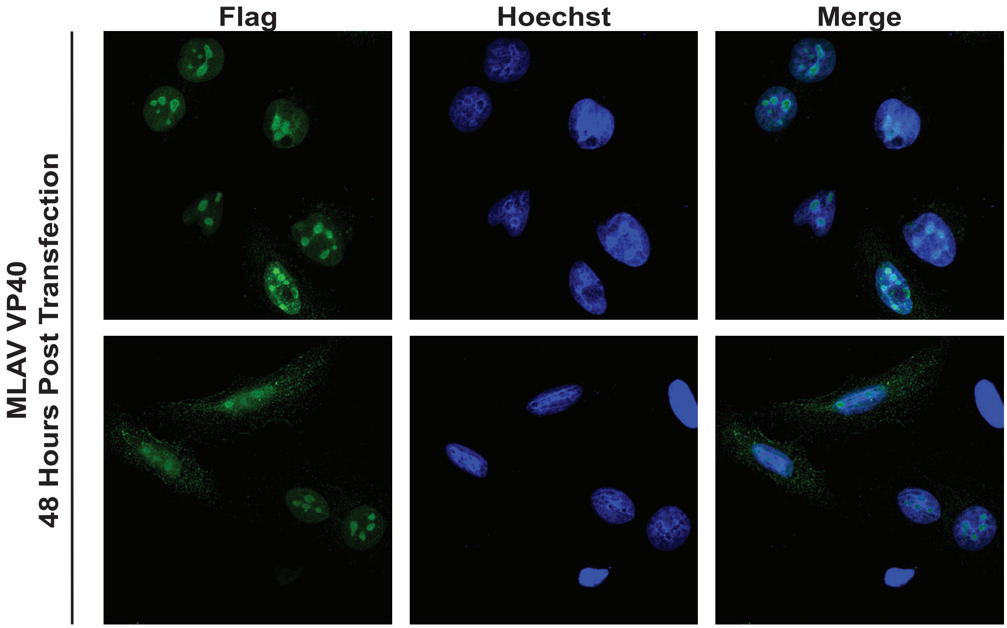

Supplemental Figure S4. Additional images of MLAV VP40 localization. Immunofluorescence confocal images to assess localization of Flag-tagged MLAV VP40 (Green) at 48 hours post transfection of HeLa cells. Hoechst (blue) staining indicates nuclei.

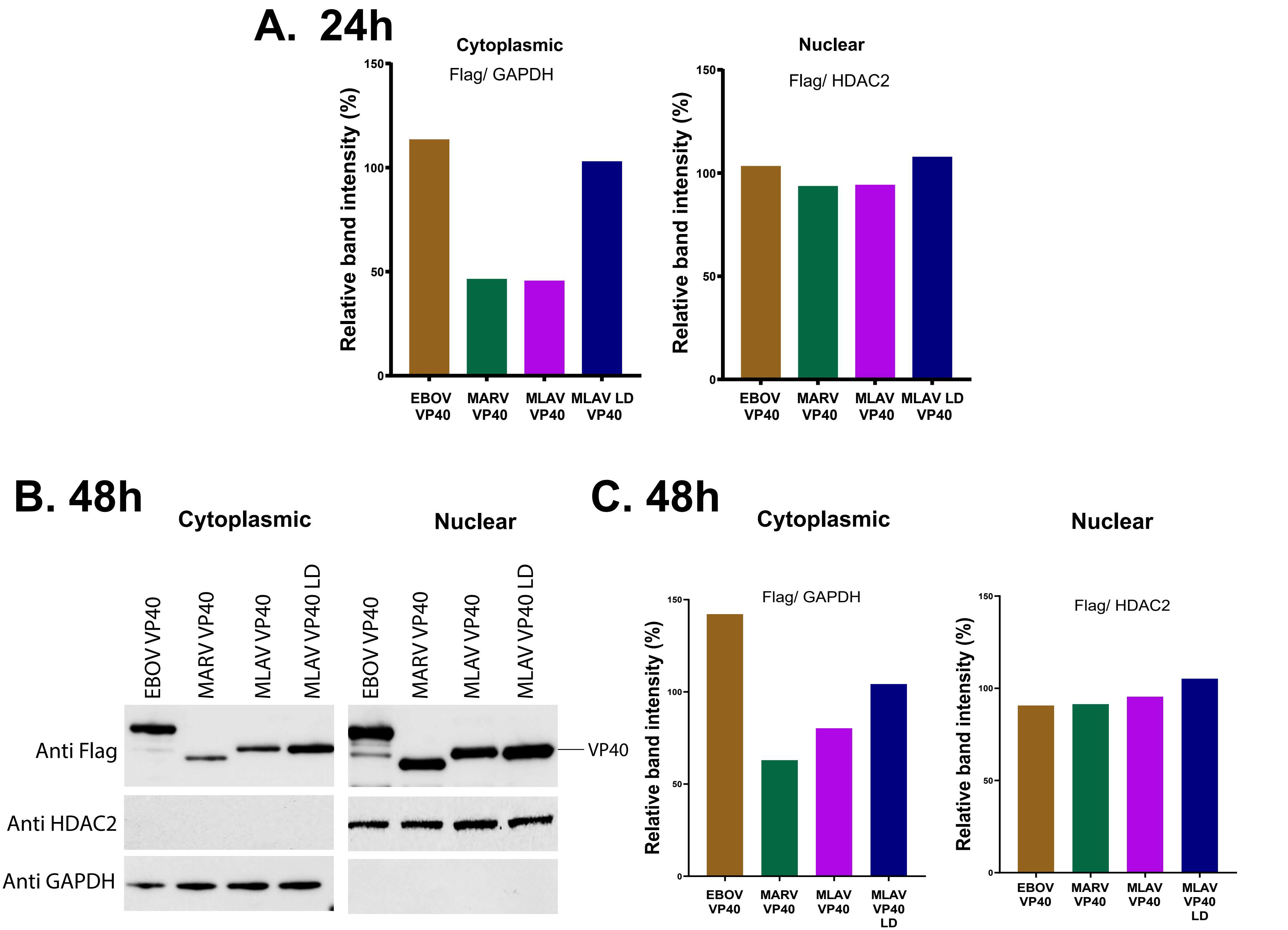

Supplemental Figure S5. Cell fraction analysis. A. Relative expression levels of proteins from Figure 6 western blots. The ratio of the chemiluminescence signal of each Flag-tagged protein to GAPDH was deter-mined for cytoplasmic fractions, and the intensity ratio of the chemiluminescence signal of each Flag-tagged protein to HDAC2 was determined for nuclear fractions. The resulting value was multiplied by 100 to express it as a percentage. B. Cells were transfected with expression plasmids for Flag-tagged EBOV VP40, MARV VP40, MLAV VP40 and MLAV VP40 LD for 48 hours. Viral proteins were detected with anti-Flag antibody. HDAC2 expression served as a control for nuclear fractions and GAPDH expression served as a control for cytoplasmic fractions. C. Quantification of the VP40 proteins expressed in B, following the same approach as described for panel A.

```
EBOV VP40       MRRVILPTAPPEYMEAIYPVRSNSTIARGGNSNTGFLTPESV-----------NGDTPSN     49
MARV VP40       ------------------------MASSSNYNTYMQYLNPPPYADHGANQLIPADQLSNQQ     37
MLAV VP40       ------------------------MASSQGSSSYNQFLSPPPYPIDQSFGHYQLEAESQPN     37
                                         : :  . .:   :*.              :  :  :

EBOV VP40       PLRPIADDTIDHASHTPGSVSSAFILEAMVNVISGPKVLMKQIPIWLPLGVADQKTYSFD    109
MARV VP40       GITPNYVGDLNLDDQFKGNVCHAFTLEAIIDISAYNERTVKGVPAWLPLGIMSNFEYPLA     97
MLAV VP40       QIMPYYVGDIDFAEQHKTDVIHAFLLEATIDLIGPTEKGRKAVPAWLPLGIISNYEYPLA     97
                 : *   . ::  .:   .*  ** *** ::: .  :   * :* *****: .:  * :

EBOV VP40       STTAAIMLASYTITHFGKATNPLVRVNRLGPGIPDHPLRLLRIGNQAFLQEFVLPPVQLP    169
MARV VP40       HTVAALLTGSYTITQFTHNGQKFVRVNRLGTGIPAHPLRMLREGNQAFIQNMVIPRNFST    157
MLAV VP40       QTVALLLTGSYTITSFSERGQKFVRVNRNGFGISSHPLQIIRNGNQAFPQAMVIPRNFLN    157
                 *.* :: .***** *  .   : :***** * **   ***:::* ***** * :*:*

EBOV VP40       QYFTFDLTALKLITQPLPAATWTDDTPTGSNGALRPGISFHPKLRPILLPNKSGKKGNSA    229
MARV VP40       NQFTYNLTNLVLSVQKLPDDAWRPSKDKLIGNTMHPAVSIHPNLPPIVLPTVKKQAYRQH    217
MLAV VP40       GNFTFQLSNIAVNIQRLPDDAWRPSKDKVVGTSMHPAIVVNPYLPPIVLPPVKKHAQKQA    217
                  **::*: : :  * **  :*   .. .   . :::*.: .:* * **:**  . :   ..

EBOV VP40       DLTSPEKIQAIMTSLQDFKIVPIDPTKNIMGIEVPETLVHKLTGKKVTSKNGQPIIPVLL    289
MARV VP40       KNPNNGPLLAISGILHQLRVEKVPEKTSLFRISLPADMFSVKEGMMKKRGENSPVVYFQA    277
MLAV VP40       KTPQAGSLLAISNLLHQLIVKKVPEKVGLFKIELPSNIFSQREGMLRRGAAIAPTVYFQA    277
                .  .   : **   *::: :  :  . .:: *.:*  :.    *          * : .

EBOV VP40       PKYIGLDPVAPGDLTMVITQDCDTCHSPASLPAVIEK 326
MARV VP40       PENFPLNGFNNRQVVLAYA--------NPTLSAV--- 303
MLAV VP40       PENMPLGGFNNRQVVMAYA--------NPSLQTTM-- 304
                *: : *. .   ::.:. :          :* :.
```

Supplemental Figure S6. Alignment of EBOV, MARV and MLAV VP40 amino acid sequences. Clustal Omega was used to align the EBOV VP40 (AAD14583.1), MARV VP40 (ABA87126.1) and MLAV VP40 (AZL87825.1) amino acid sequences. Blue highlight, residues conserved among all three VP40s. Green highlight, residues conserved between MARV and MLAV VP40s. Yellow highlight, residues conserved between EBOV and MARV VP40s. PPXY late domain motifs are indicated with bold text.
